# Functionalized Multi-Wall Carbon Nanotubes Enhance Transfection and Expression Efficiency of Plasmid DNA in Fish Cells

**DOI:** 10.3390/ijms17030335

**Published:** 2016-03-03

**Authors:** Guanglu Liu, Yuan Wang, Yang Hu, Xiaobo Yu, Bin Zhu, Gaoxue Wang

**Affiliations:** 1 College of Science, Northwest A & F University, Xinong Road 22nd, Yangling 712100, China; 563310592@163.com (G.L.); 18729316235@163.com (Y.H.); 2 College of Animal Science and Technology, Northwest A & F University, Xinong Road 22nd, Yangling 712100, China; 18792426961@163.com (Y.W.); yuxiaobo6@126.com (X.Y.); zhubin1227@126.com (B.Z.)

**Keywords:** DNA vaccine, multi-wall carbon nanotubes (MWCNTs), grass carp reovirus (GCRV), outer capsid protein VP5

## Abstract

DNA vaccines are considered to be the most promising method against infectious diseases in the aquaculture industry. In the present study, we investigated the potency of ammonium group-functionalized multi-walled carbon nanotubes (MWCNTs) in enhancing the transfection and expression efficiency of plasmid DNA (pEGFP-*vp5*) in *Ctenopharyngodon idellus* kidney (CIK) cells. Agarose gel shift assay results show that ammonium group-functionalized carbon nanotubes are able to condense DNA in varying degrees. Scanning electron microscope (SEM) images shows that CIK cells show a great affinity for MWCNTs-NH_3_^+^ and the CNTs covering the cell surface tend to orient their tips perpendicularly to the cell surface, and appear to be “needle-pricking the cells”. Transmission electron microscope (TEM) images confirmed that MWCNTs-NH_3_^+^ penetrate the cell membranes and are widely dispersed in the CIK cell. Real-time PCR was used to detect the transfection efficiency through the expression of the outer capsid protein (VP5). The results showed that the MWCNTs-NH_3_^+^:DNA complexes are able to transfect CIK cells effectively at different charge ratio than naked DNA. Subsequent studies confirmed that both functional groups and charge ratio are important factors that determine the transfection efficiency of plasmid DNA. All these results indicated that MWCNTs-NH_3_^+^:DNA complexes could be suitable for developing DNA vaccine for the control of virus infection in the aquaculture industry.

## 1. Introduction

During the past decades, a continuous growth in the aquaculture industry is emerging worldwide, with ever increasing cultured fish production from marine and freshwater environments. Among the cultured fishes, the most significant growth has been seen in carp production, such as grass carp, which are farmed in intensive conditions. However, any intensive bio-production, whether on land or at sea, will experience disease problems [1]. Among the problems that the aquaculture industry has to address, infectious pathogens are the most important [2,3,4]. In 1993, the economic losses caused by the infectious pathogens are more than ten percent of the total value of the fishery industry [5], and the loss ratio is increased gradually in the last decades. In 2010, aquaculture in China suffered production losses of 1.7 million tons caused by infectious pathogen diseases [6]. Hence, economical and environmentally-friendly methods which can prevent or control infectious diseases are essential to the continued development of aquaculture.

At present, chemicals and antibiotics have been used frequently in aquaculture to control bacterial or parasitic diseases and partially solved the problem [7]. For viral infections, where there are no treatments available, the outbreak of disease in facilities usually requires destruction of the infected quotient before starting anew [2,8]. Moreover, treatment with chemical agents and antibiotics frequently and excessively will inevitably lead to drug resistance, and drug residues in fish or in the aquatic environment have also raised concerns [9]. The successful application of vaccines in intensive fish culture provides an effective strategy in treatment of infectious diseases. Basically, there are four types of vaccines: live, attenuated or inactivated vaccines, purified subunits of the pathogen (such as proteins or glycoproteins), and DNA vaccines [10]. The DNA vaccine consisting of naked plasmid DNA is usually delivered intramuscularly, leading to the gene expression of pathogenic proteins in the muscle tissue of the vaccinated fish. This strategy was considered to be the most effective against infectious diseases. Three routes of gene vaccine immunization are applied including intramuscular injection, oral administration and immersion of the vaccine [11,12,13]. However, the former two vaccine therapies are largely limited by the high cost, poor efficiency, as well as serious side effects such as regional variability. The immersion therapy of optimized DNA vaccines in aquaculture has been proven to be apparently effective to gain high immunity in aquatic organisms. Nevertheless, the uptake of optimized DNA through immersion remains to be a bottleneck in the application, thus prompting the development of an efficient DNA carrier.

Carbon nanotubes (CNTs) were first discovered in 1991, since their discovery, carbon nanotubes have attracted considerable attention because of their unique physical, mechanical, biologic and electronic properties [14]. CNTs have been widely applied in various fields such as field emission [15], energy storage [16], molecular electronics [17], and atomic force microscopy (AFM) [18]. Meanwhile, CNTs have effectively been employed to deliver biologically active cargo into living systems for the purposes of disease diagnosis and therapy in biological and medical fields [19]. Previous studies have shown that functionalized carbon nanotubes could provides multiple sites for the binding of drugs, amino acids, sugars, oligonucleotides, peptide nucleic acids or proteins through non-covalent bonds or stable covalent bonds [20,21,22,23,24]. Pantarotto *et al.* (2004) [25] reported that peptide functionalized carbon nanotubes are capable of penetrating the mammalian plasma membranes and translocated to the cell nucleus. They further demonstrated that *f*-SWNT/DNA can lead to 5–10 times higher levels of gene expression than treatment of Chinese hamster ovary cells with naked DNA alone [26]. Another study confirmed that cationic carbon nanotubes are able to condense DNA to varying degrees and the electrostatic complex formation between *f*-CNTs with DNA exhibited upregulation of marker gene expression over naked DNA using a mammalian (human) cell line [27]. However, utilizing the carbon nanotubes as DNA vaccine carriers in aquatic animal cell lines were scarcely mentioned in relevant research.

Grass carp reovirus (GCRV), as a representative malignant virus in cyprinidae [28], was selected as the subject of our research investigation. The GCRV protein (VP5) is an important outer capsid protein that plays an important role during viral infection, such as recognition and attachment to receptors in the host cell surface, as well as penetration into the host cell membrane during virus assembly [29]. Therefore, in the present work, we constructed a pEGFP-*vp5* plasmid (pDNA) that includes the gene fragment encoding VP5 and EGFP protein as the candidate for DNA vaccine and four types of amino-functionalized MWCNTs (MWCNTs-NH_3_^+^) were prepared as carriers for plasmid DNA. By varying the MWCNTs-NH_3_^+^:DNA charge ratio and distinct chemical modification of the nanotubes surface, we further investigated how these two factors influence the transfection efficiency in *Ctenopharyngodon idellus* kidney (CIK) cells. Our study provides a fundamental basis for the potential application of CNTs-DNA vaccine delivery system in controlling infectious disease in the aquaculture industry.

## 2. Results

**Figure 1:**
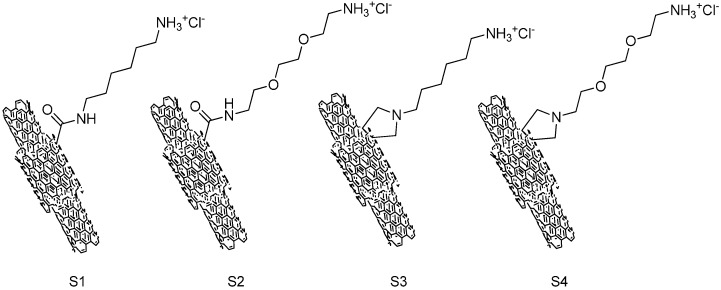
Molecular structures of ammonium-functionalized MWCNTs.

The structure of ammonium-functionalized MWCNTs is shown in Figure 1. The carboxylic-functionalized MWCNTs were covalently modified using a method based on the 1,3-dipolar cycloaddition of azomethineylides or amidation reaction to obtain the ammonium-functionalized MWCNTs (S1–S4). S3 and S4 were functionlized with a pyrrolidine ring bearing a free amino-terminal oligoethylene glycol or *N*-hexyl moiety attached to the nitrogen atom; S1 and S2 were obtained by using 1,2-*bis*(2-aminoethoxy)ethane or hexamethylenediamine react with MWCNTs-COCl directly through an amidation reaction.

**Figure 2:**
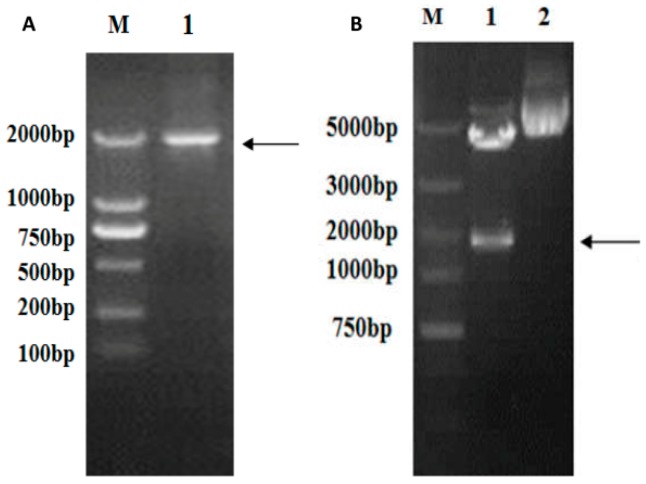
Verification of the *vp5* gene. (**A**) RT-PCR amplification of *vp5*: lane M, DNA marker; lane 1, *vp5* gene; (**B**) Analysis of the recombinant plasmid: lane M, DNA marker; lane 1, double enzymes digested pEGFP-*vp5* with *Nhe* I and *Xho*I; lane 2, pEGFP-*vp5*. Black arrow: the *vp5* gene stripe.

The full-length *vp5* gene (size 1981 bp) was amplified through PCR using specific primer pairs from the viral genome cDNA mix, as can be seen in Figure 2A. The *vp5* gene segment was cloned into the eukaryotic expression plasmid pEGFP-c1 to generate pEGFP-*vp5*. It was identified by restriction enzyme digestion (Figure 2B) and sequenced to analyze its base composition. The abovementioned results confirmed that recombinant expression plasmid pEGFP-*vp5* was successfully constructed.

**Figure 3:**
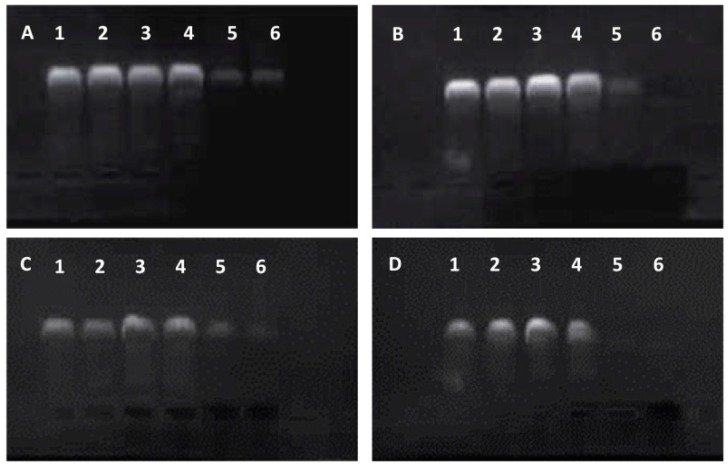
Electrophoretic mobility of *f*-MWCNTs: DNA complexes (**A**: S1:DNA; **B**: S2:DNA; **C**: S3:DNA; **D**: S4:DNA). In all panels, lane 1 represents 0.3 µg of free DNA. All other lanes contain *f*-MWCNTs complexes to 0.3 µg of DNA at various charge ratios: lane 2, 1:1; lane 3, 2:1; lane 4, 4:1, lane 5, 6:1, lane 6, 8:1.

Subsequently, the migration of the f-MWCNT:DNA complexes was investigated by agarose gel electrophoresis and the degree of DNA condensation by ethidium bromide exclusion (see Figure 3). The highly purified pEGFP-*vp5* plasmid DNA was added in lane 1 as blank control (lane 1, Figure 3). When the plasmid DNA condensed on the MWCNTs-NH_3_^+^ surface, the complex may have excluded ethidium bromide intercalation and quenched the fluorescence signal. Therefore, it was not likely to observe condensed DNA participating in the MWCNTs-NH_3_^+^:DNA complexes. In general, the fluorescent bands observed in Figure 3 emanate from free (uncomplexed) plasmid DNA which allows for ethidium bromide intercalation. As shown in Figure 3, the fluorescence intensity of the free plasmid bands with increasing MWCNTs-NH_3_^+^:DNA charge ratio was decreased significantly, due to reduced free DNA bases for ethidium bromide intercalation caused by the higher degree of DNA condensation. In the case of four types of complexes, a bright fluorescent band is observed for the complex with 1:1 charge ratio (lane 2 in Figure 3A–D), indicating the presence of a large amount of free DNA. In our case, this was possible only in the case of S1 (Figure 3A), which seems to be incapable of condensing plasmid DNA completely. In lanes 2–6, the fluorescence signal of the free plasmid DNA is clearly observed. In Figure 3, a remarkable decrease in the fluorescence intensity was observed when the MWCNTs-NH_3_^+^:DNA charge ratio is increased to 6:1, but little difference is observed when the charge ratio is further increased to 8:1.

**Figure 4:**
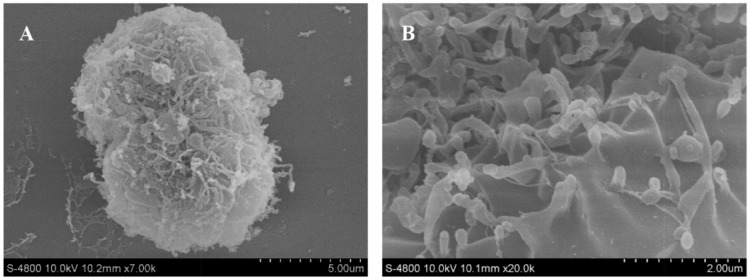

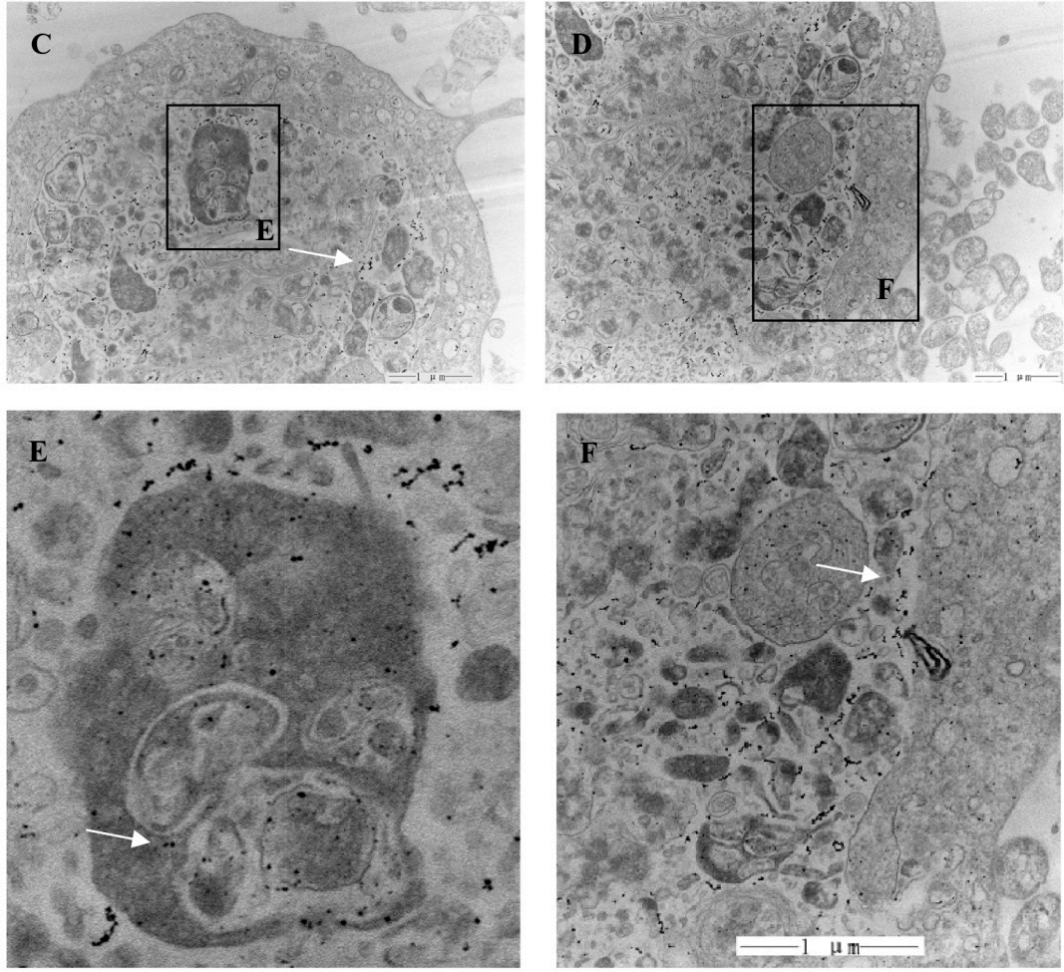
SEM and TEM images of CIK cell treated with MWCNTs-NH_3_^+^:DNA (S4:DNA). (**A**–**B**) CIK cell interacting with S4:DNA complexes; (**C**–**F**) ultrathin transverse section of CIK treated with S4:DNA complexes. MWCNTs-NH_3_^+^ is marked by white arrow.

In addition, the interaction of theMWCNTs-NH_3_^+^:DNA complexes with CIK cells was examined by SEM and TEM, as depicted in Figure 4. CIK cells were incubated with ammonium functionalized MWCNTs S4 at a concentration of 20 μg/mL. Figure 4 shows CIK cells incubated with MWCNTs-NH_3_^+^:DNA complexes. The interaction of MWCNTs-NH_3_^+^ with the cell surface is shown in the SEM images (Figure 4A,B). SEM images indicated that the CIK cells show a great affinity for MWCNTs-NH_3_^+^. Figure 4A exhibited a dividing CIK cell covered by MWCNTs-NH_3_^+^. While most of the MWCNTs-NH_3_^+^ covering the cell surface tend to orient their tips perpendicularly to the cell surface, and appear to be ‘‘needle-pricking the cells’’, (see Figure 4B). Upon interaction with CIK cells, these *f*-MWCNTs penetrate the cell membranes and disperse into the cells. As shown in Figure 4C, a considerable amount of nanotubes were clearly traced inside the cell. Subsequent magnifications (Figure 4D–F) exhibit a higher-resolution horizon of the intracellular localization of the MWCNTs-NH_3_^+^. Detailed analysis of the cell sections also permits the observation of nanotubes in the process of crossing the plasma membrane barrier.

**Figure 5:**
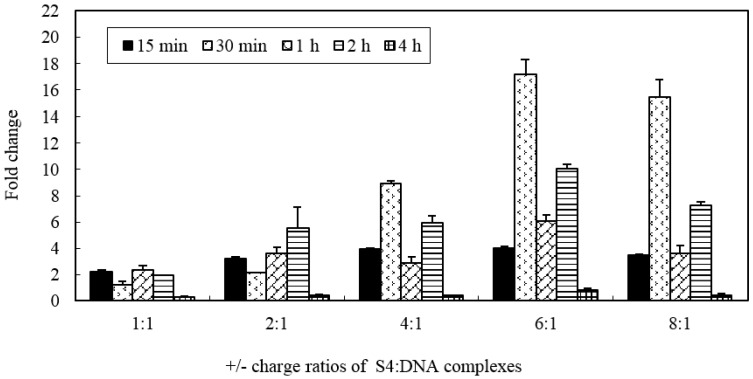
Expression of *vp5* gene in CIK cells following gene delivery with S4: DNA complexes at various charge ratios. Each MWCNTs-NH_3_^+^:DNA charge ratios were tested with five different incubation time periods groups. Data are means for three assays and presented as the means ± standard error.

In order to assess whether the MWCNTs-NH_3_^+^ has the ability to create temporary transmembrane ‘‘nanochannels’’ for simultaneous particle delivery, S4:DNA complexes with various charge ratios were chosen for investigation. The ability of the MWCNTs-NH_3_^+^ delivery plasmid DNA to the cell was evaluated by gene expression level. Figure 5 shows the levels of marker gene (*vp5*) expression in CIK cells after exposure to nanotubes connected to plasmid DNA encoding the gene. As shown in Figure 5, we can see that all of the different charge ratio complexes are able to transfect CIK cells better than naked DNA. No cytotoxicity was observed in this study (the highest nanotubes concentration used in this cytotoxicity test was 0.2 mg/mL), even when the MWCNTs-NH_3_^+^ were incubated with the CIK cells for 4 h in this research. The level of gene expression confirms that the S4/DNA complexes charge ratios between 2:1 and 8:1 led to 3–17 times higher than treatment of the CIK cells with DNA alone. In this study, we observed an increase in gene expression with increasing charge ratios for S4:DNA complexes that resulted in optimum gene delivery capacity (*i.e.*, between 1:1 and 6:1). The highest gene expression level was observed after a 30-min incubation period with 6:1 charge ratio S4: DNA complexes (more than 17 times higher than the naked DNA group). Interestingly, compare to the 6:1 charge ratio, the gene expression level appears to slightly decrease when the charge ratio increased to 8:1. These results may be an indication that the charge ratio of the MWCNTs-NH_3_^+^ and plasmid DNA may play a considerable factor in the level of gene expression.

**Figure 6:**
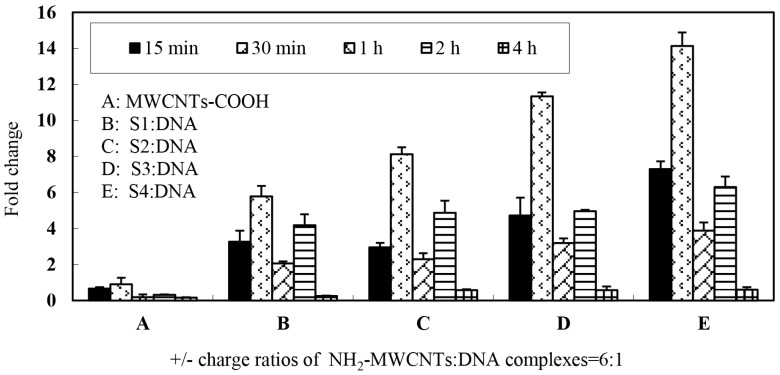
Expression of *vp5* gene in CIK cells following gene delivery with four types of MWCNTs-NH_3_^+^:DNA complexes at stationary charge ratios (6:1). Stationary charge ratios of each types of MWCNTs-NH_3_^+^:DNA were tested with five different incubation time periods groups (A–E). Data are means for three assays and presented as the means ± standard error.

Furthermore, we monitored the gene expression level of which carbon nanotubes surface were functionalized by different structure of ammonium-group. Figure 6 shows the levels of marker gene (*vp5*) expression in CIK cells after exposure to different types of nanotubes connected to plasmid DNA encoding the gene. In this portion, 6:1 charge ratio was selected for each type of MWCNTs-NH_3_^+^ and plasmid DNA complexes. The data demonstrate that four types of complexes are able to transfect CIK cells with greater efficiency than naked DNA and S4 appear to be most efficient at gene transfer when complexed to plasmid DNA at the 6:1 charge ratio. As shown in Figure 6, it should be noted that a regular variation trend of the gene expression level was observed with increasing incubation times in all of the four groups. The first peak of gene expression was observed in the 30-min incubation period group, the gene expression level was 6–14 times higher than treatment of cells with DNA alone. The gene expression significantly declined after a 1-h incubation period and a second peak of gene expression was observed in the 2-h incubation group. Most interestingly, the S1 and S2 MWCNTs-NH_3_^+^ exhibited a significant difference in gene expression levels, however there was only a slight difference in the ammonium group structures. These results indicated that the structure of ammonium groups on the carbon nanotubes surface may play an important role while interacting with plasmid DNA as DNA vaccine.

## 3. Discussion

GCRV, considered to be the most serious pathogen threatening grass carp production with high mortality in China, is a member of the *Aquareovirus* genus in the family *Reoviridae* [30,31]. Nowadays, whole virus-inactivated vaccine is used in aquaculture for prevention of the infectious disease and the traditional methods to prevent the disease utilize the whole virus-inactivated vaccine through intramuscular injection method or immersion bath method, but is limited by the unsatisfactory immunizing potency or high labor cost [32]; thus the aforementioned methods cannot be widely used in the grass carp aquaculture industry. DNA vaccines serve as a potential alternative to the conventional inactivated vaccines with practical and immunological advantages, and have gained increasing interest in aquaculture. They are also regarded as genetic or polynucleotide vaccines that represent a novel approach for achieving specific immune activation [33]. Extensive studies have shown that delivery of naked DNA into mammalian cells could bring about high gene expression level [25,27,34]. Therefore, gene vaccines may be a better choice against GCRV.

Previous studies of GCRV structures reveal that VP5 and VP7 are the outer capsid proteins of the virus [35]. VP5 is reported to play a essential function in viral infection, such as recognition and attachment to receptors in the host cell surface, as well as penetration into the host cell membrane during the virus assembly [29]. Therefore, in our research, a plasmid DNA contains VP5 and EGFP protein gene segment was constructed as a DNA vaccine candidate.

CNTs have effectively been employed, in biological and medical aspects, to deliver bioactivators into living creature for the purposes of disease diagnosis and therapy [36]. The functionalized carbon nanotubes, such as ammonium or carboxyl-functionalized carbon nanotubes, can provides multiple sites for the attachment of drugs, amino acids, proteins and other substances that would not otherwise be taken up, and with no apparent side effects [37,38,39]. Numerous studies indicated that complex formation between *f*-CNTs and plasmid DNA can constitute a novel class of non-viral gene delivery systems. In this study, we utilize the physicochemical interactions between cationically-modified multi-wall carbon nanotubes and pEGFP-*vp5* plasmid DNA constructs a novel carbon nanotubes-based gene-transfer vector systems. However, for the purpose of developing a *f*-CNTs:DNA gene delivery system, the principles of rational design, in particular, optimization of charge ratio and structure of cargo-ship, must be premeditated in this study [40].

Extensive studies have focused on the *f*-SWCNTs for intracellular delivery applications in mammalian and bacterial cells, such as HeLa cells, adenocarcinoma lung cells and *E. coli*, while aquatic cells were scarcely investigated. In order to determine whether it is possible to use *f*-MWCNTs for intracellular delivery applications in fish cells we studied their interaction with CIK cells; the interaction of the *f*-MWCNTs with cells was studied using SEM and TEM. SEM imaging indicated that carbon nanotubes with hydrophilic functional groups can spontaneously insert into a lipid bilayer, and appear to be ‘‘needle-pricking the cells’’. The TEM imaging shows that plenty of nanotubes are clearly visible inside the CIK cell (Figure 4). That result proves that the f-MWCNTs are possible carriers for gene delivery in fish cells.

Part of cation- or polycation-DNA complexes is capable of transfection *in vitro* at a different charge ratios and the transfect-efficiency may greatly increase when the charge ratio is optimized. There are many factors, such as surface charge, complex dimensions, DNA topology, and degree of condensation, that may play crucial roles in gene transfer [41,42,43]. In order to assure that the *f*-CNT:DNA complexes are capable for gene delivery, we need to confirm the following three aspects: Firstly, the plasmid DNA can be condensed by the nanotubes; Secondly, the complexes can be delivered into the target cell; Thirdly, the plasmid DNA could detach from the nanotube and enter the nucleus prior to transgene expression. The mobility shift experiments were employed to examine the DNA conformation and the efficiency of condensation influenced by the *f-*MWCNT and the overall motility characteristics of the *f-*MWCNT:DNA complexes. Assuming that the plasmid DNA was absorbed by the *f-*MWCNT, ethidium bromide was excluded from the condensed plasmid DNA, making it impossible to directly monitor the migration of an *f-*MWCNT: DNA complex. Otherwise, the DNA was visible on the gel corresponded to free DNA rather than DNA involved in forming the *f-*MWCNT complex, we were able to indirectly monitor the formation of the complex. As sizeable amounts of DNA are condensed, the amounts of free DNA rarely exist, therefore weaken the corresponding fluorescence signal. Figure 3 shows that with the increasing charge ratio, the fluorescence intensity in all of the four types of MWCNTs-NH_3_^+^ lanes rapidly declined, indicating the formation of tightly packed complexes with the DNA. As shown in Figure 3A, the gel images shows that pieces of free DNA are present even at the 8:1 charge ratio, indicating that S1 were weakly bound to DNA complexes than others even at high charge ratios.

Gene delivery and expression studies based on S4 demonstrate that each charge ratio of the S4: DNA complexes are able to mediate enhanced gene transfer over plasmid DNA alone. The data from RT-PCR analysis confirmed that the gene expression level was gradually enhanced with increasing charge ratio. Interestingly, the RT-PCR data showed that exorbitant charge ratio may decrease the expression level. It appears that optimal gene-transfer efficiency when using *f-*MWCNT:DNA complexes may occur when DNA is only partially condensed. We repeated those studies herein using four types of carbon nanotubes to confirm that all four types of carbon nanotubes are able to mediate heightened gene transfer over plasmid DNA alone. However, the present results clearly demonstrated that *f*-CNT modified with different amino side chains, such as hexamethylenediamine, leading to various gene expression levels. This implies that the binding stability of *f-*CNT:DNA complexes were influenced directly by the amino side chains variances. Owing to long-term pharmacological applications of such *f-*CNT:DNA systems, to avoid the dissociation on dilution or competition with other molecules (such as blood components and protein), a forceful electrostatic interaction is necessary between the plasmid and carbon nanotubes. However, if DNA is too tightly complexed, it may be unable to detach from the nanotubes, therefore leading to compromised gene expression. Therefore, determination of a reasonable balance between DNA condensations, tight association with the *f*-CNT surfaces is essential to develop their capacity as novel gene delivery systems.

## 4. Materials and Methods

### 4.1. Materials

MWCNTs (OD: <8 nm; Length: 10–30 µm; Purity: >95%) were purchased from Chengdu Organic Chemicals Co., Ltd., Chinese Academy of Sciences (Chengdu, China). Sulfuric acid and nitric acid was purchased from Sinopharm Chemical Reagent Co., Ltd. (Shanghai, China). All other organic reagents used were purchased from Sigma (St. Louis, MO, USA), and used as received.

### 4.2. Amination Modification of MWCNTs

Figure 1 shows the structure of four types of ammonium functionalized multi-wall carbon nanotubes (S1–S4). The synthesis of S1–S4, as well as preparation and purification of the mono-protected diamines compounds, has been previous described [44,45,46]. Briefly, pristine MWCNTs was purified by refluxing with 2 M HCl for 12 h to remove the amorphous carbon particles and metallic catalyst and then suspended in a concentrated mixture acid (H_2_SO_4_:HNO_3_ = 3:1, *v*/*v*) at room temperature for 24 h with vigorous stirring to obtain the MWCNTs-COOH. The MWNTs-COCl was obtained by refluxing MWCNTs-COOH with thionyl chloride for 6 h. S1 and S2 were synthesized by stirring MWNTs-COCl with mono-protected diamines in dry dimethyl formamide [44]. S3 and S4 were obtained based on chemical modification of the side wall through 1, 3-dipolar cycloaddition of azomethineylides [45,46]. The BOC-protecting group was removed from the functionalized MWCNTs by stirring with 4 M HCl in CH_2_Cl_2_ for 4 h [47]. Then the mixture was filtered and the solid dried at 60 °C to obtain the amino-functionalized MWCNTs. Each type of MWCNTs-NH_3_^+^ was dissolved in deionized water at a concentration of 1 mg/mL. All solutions were sonicated for 30 min at room temperature in an ultrasonic bath until dispersed throughout an aqueous phase and then stored at −20 °C until needed.

### 4.3. Construction of a Donor Vector pEGFP-vp5

The GCRV strain used in this test was obtained from an infected grass carp in fish farm (Rougu, Shaanxi, China) and stored in our laboratory [48]. QIAGEN Viral RNA Mini Kit (Qiagen, Duesseldorf, Germany) was used to extract the viral genomic RNA and the RNA was converted to cDNA with RNA PCR KitVer.3.0 (Qiagen, Germany) based on the manufacturer’s instructions. Using primer pairs consisting of GCRV-F (5′-CGCGCTAGCATGTGGAACGTTCAAACCT-3′ the underline indicates the *Nhe* I site) and GCRV-R (5-CCGCTCGAGTCACTTGCCGGGCCACAAGCTC-3′ the underline shows the *Xho*I site), the full-length *vp5* gene was amplified through PCR. The gene segment was gel-purified, cloned into pMD19-T vector (Takara, Dalian, China), transformed into *Escherichia coli* DH5α (*E. coli* DH5α) competent cells (Beijing CoWin Biotech Corp, Beijing, China) and sequenced by Sangong Biological Company (Shanghai, China), then the *vp5* gene was excised from pMD19T-*vp5* by digestion with *Nhe* I/*Xho*I was ligated into the pEGFP TM Dual (Invitrogen, Carlsbad, CA, USA) to generate pEGFP-*vp5*. Then the recombinant plasmid pEGFP-*vp5* was transferred into *E. coli* DH5α competent cells and identified by restriction enzyme digestion and PCR amplification, simultaneously. Besides, the recombinant cassette, which contains the objective gene was also verified by Sangong Biological Company (Shanghai, China). The positive clone was screened in selective plates of ampicillin and then incubated in incubator (37 °C, 180 r/min, 10 h). The Endo-free Plasmid Mini Kit (Omega, Norcross, GA USA) was used to isolate the recombinant plasmid DNA and the concentration of plasmid DNA was measured by the NanoDrop spectrophotometer (ND-1000, NanoDrop Technologies Inc., Wilmington, DE, USA) at 260 nm. Then the plasmid DNA was hydrated in sterile water at a concentration of 1000 μg/mL and the liquors were stored frozen at −80 °C until needed.

### 4.4. MWCNTs-NH_3_^+^:DNA Complexes

To prepare the MWCNTs-NH_3_^+^:DNA complexes, plasmid DNA (1.2 μg) solution were mixed with each type of MWCNTs-NH_3_^+^ based on the MWCNTs-NH_3_^+^:DNA charge ratio (1:1, 2:1, 4:1, 6:1 and 8:1). The mixture was diluted to 4 mL by using the minimum essential medium (MEM) and mixed by rapidly pipetting 10–20 times, yielding a final DNA concentration of 0.3 μg/mL. Furthermore, maximum concentration of MWCNTs-NH_3_^+^ solution (equivalent to 8:1 charge ratio) was chose as nanotubes-only control and a 0.3 μg/mL DNA solution was prepared as plasmid-only control simultaneously. This process was repeated three times for each charge ratio tested. Complexes were allowed to form for 40 min at room temperature before use.

### 4.5. Electrophoretic Motility Shift Assay

The different charge ratios of MWCNTs-NH_3_^+^:DNA complexes, which consists of plasmid DNA (0.3 μg) and different-dose of carbon nanotubes was added to a 1% agarose gel in Tris-acetate-EDTA buffer containing ethidium bromide. Meanwhile a sample that contained only 0.3 μg of free DNA, was prepared as control group. The gel was photographed under UV light using a UVP gel documentation system (Upland, CA, USA) after being run for 15 min at 120 V. Each sample was tested three times.

### 4.6. Preparation of Cell Sections for SEM and TEM Analysis

Ultrastructural changes of MWCNTs-NH_3_^+^/DNA complex incubated CIK cells are observed using scanning and transmission electron microscopy, respectively. The procedure for SEM and TEM sample were prepared as described previously [49,50]. Briefly, CIK cells were cultured in MEM medium contain 10% FBS in a 6-well plate at 28 °C in the present of 5% CO_2_ until 90% confluency was reached. The cells were then incubated with a solution of MWCNTs-NH_3_^+^:DNA complexes (the dose of MWCNTs-NH_3_^+^ = 20 μg/mL) in MEM medium for 30 min, washed twice with MEM medium. As an SEM sample, the cells were fixed with 2.5% glutaraldehyde at 4 °C overnight. Thereafter the samples were washed with PBS 3 times, dehydrated in a series of ethanol solutions, passage through acetone, isoamyl acetate and completely dried with a critical-point dryer. Then the prepared samples were sputter-coated with gold-palladium. The sample was observed and photographed with a scanning electron microscopy (SEM-6360 LV, Hitachi, Japan) at 10 kV. As a TEM sample, the incubated cells was washed three times with PBS (15 min each wash) and then fixed with glutaraldehyde solution (2.5%) and osmium tetroxide, dehydrated and embedded. Ultrathin section was cut with a microtome and stained with uranyl acetate and lead citrate; the samples were characterized by transmission electron microscope (TEM-HT7700, Hitachi, Japan).

### 4.7. RT-PCR Detection of the Expression of vp5 in CIK Cells

CIK cells were cultured in MEM containing 10% FBS (Invitrogen/Gibco, Paisley, UK) until just confluent in 6-well plates. Complexes were formed by diluting 1.2 µg of pEGFP-*vp5* and the appropriate amount of S1, S2, S3 or S4 in 4 mL of MEM to yield the indicated charge ratios. The DNA solution was mixed with *f*-MWCNTs by rapid pipetting and allowed to stabilize for 40 min. Then the complete media were removed from the 6-wells plate and replaced with 4 mL of the various MWCNTs-NH_3_^+^/DNA complexes. Cells treated with naked DNA or MWCNTs-NH_3_^+^, were used as control group. Before the transfection medium was removed and replaced with complete medium, the cells were incubated with the complexes for 30 min at 28 °C. CIK cells were selected and washed with PBS for 15 min, 30 min, 1, 2 and 4 h, and conserved at −80 °C for RT-qPCR analysis. TRIzol reagent (Invitrogen, Carlsbad, CA, USA) was used to extracted the total RNA and then the total RNA was incubated with RNase-free DNase I (Life Technology, Gaithersburg, MD, USA) and random hexamer primers and M-MLV Reverse Transcriptase (Promega, Madison, WI, USA) was used before being reversely transcribed into cDNA in order to eliminate contaminated genomic DNA which might disturb the PCR reactions. The quantitative RT-PCR (qRT-PCR) was used to determine the expression of recombinant plasmid in CIK cells. PCR was carried out with specific primers G-F1 (5′-CGCGCTAGCATGTGGAACGTTCAAACCT-3′) and G-R1 (5′-CCGCTCGAGTCACTTGCCGGGCCACAAGCTC-3′). The 18S gene was used as an internal control (5′-ATTTCCGACACGGAGAGG-3′; 5′-CATGGGTTTAGGATACGCTC-3′).

### 4.8. Statistical Analysis

Statistical analysis was performed using the SPSS Version 11.0 software package (SPSS Inc., Chicago, IL, USA). mRNA relative expression was calculated using ΔΔ*C*_t_ method [51]. Statistical significance of the differences between the experimental groups was tested using one-way ANOVA followed by Tukey’s test.

## 5. Conclusions

In conclusion, the present study shows that amino-modified MWCNTs:DNA are capable of transfecting CIK cells *in vitro*, and the exogenous gene (*vp5*) can be expressed normally in CIK cells with high gene expression level than naked plasmid DNA. The ability of *vp5* gene expression level were affected by the fine distinction in amino side chains and charge ratio of the *f*-MWCNTs:DNA complexes. Based on the study will allow the implementation of rational design strategies in the development of *f*-MWCNTs:DNA complexes as effective DNA vaccines to prevent virus infection in the fishery industry.
